# Inulin-grown *Faecalibacterium prausnitzii* cross-feeds fructose to the human intestinal epithelium

**DOI:** 10.1080/19490976.2021.1993582

**Published:** 2021-11-18

**Authors:** Raphael R. Fagundes, Arno R. Bourgonje, Ali Saeed, Arnau Vich Vila, Niels Plomp, Tjasso Blokzijl, Mehdi Sadaghian Sadabad, Julius Z. H. von Martels, Sander S. van Leeuwen, Rinse K. Weersma, Gerard Dijkstra, Hermie J. M. Harmsen, Klaas Nico Faber

**Affiliations:** a Department of Gastroenterology and Hepatology, University of Groningen, University Medical Center Groningen, Groningen, The Netherlands; b Department of Genetics, University of Groningen, University Medical Center Groningen, Groningen, The Netherlands; c Department of Medical Microbiology and Infection Prevention, University of Groningen, University Medical Center Groningen, Groningen, The Netherlands; d Department of Laboratory Medicine, University of Groningen, University Medical Center Groningen, Groningen, The Netherlands; e Institute of Molecular Biology & Biotechnology, Bahauddin Zakariya University, Multan, Pakistan

**Keywords:** Gut bacteria, dysbiosis, fructose, intestinal epithelium, Faecalibacterium, inulin

## Abstract

Many chronic diseases are associated with decreased abundance of the gut commensal *Faecalibacterium prausnitzii*. This strict anaerobe can grow on dietary fibers, e.g., prebiotics, and produce high levels of butyrate, often associated to epithelial metabolism and health. However, little is known about other *F. prausnitzii* metabolites that may affect the colonic epithelium. Here, we analyzed prebiotic cross-feeding between *F. prausnitzii* and intestinal epithelial (Caco-2) cells in a “Human-oxygen Bacteria-anaerobic” coculture system. Inulin-grown *F. prausnitzii* enhanced Caco-2 viability and suppressed inflammation- and oxidative stress-marker expression. Inulin-grown *F. prausnitzii* produced excess butyrate and fructose, but only fructose efficiently promoted Caco-2 growth. Finally, fecal microbial taxonomy analysis (16S sequencing) from healthy volunteers (n = 255) showed the strongest positive correlation for *F. prausnitzii* abundance and stool fructose levels. We show that fructose, produced and accumulated in a fiber-rich colonic environment, supports colonic epithelium growth, while butyrate does not.

## Introduction

The human gut microbiota is characterized by a vast collection of bacterial species that colonize the gastrointestinal (GI) tract and plays a pivotal role in human health and disease.^1^ The gut microbiota composition is highly dynamic and complex, with high intra- and inter-individual diversity.^2,3^ A disturbed gut microbiota homeostasis is termed “dysbiosis” and, typically, characterized by a combination of increased potentially pathogenic bacteria and decreased beneficial bacterial abundances.^3,4^ Dysbiosis is associated with an increasing number of human diseases, including colon cancer, type-2 diabetes mellitus and inflammatory bowel disease (IBD).^5^ Especially in IBD, dysbiosis is illustrated by decreased bacterial diversity with significant shifts of microbial populations, e.g. decreased abundances of *Bifidobacterium* and *Faecalibacterium* and increased numbers of adherent-invasive *Escherichia coli* (AIEC) and mucin-degrading *Ruminococcus gnavus*.^5–8^ A consistent observation is the decreased abundance of the strictly anaerobic, butyrate-producing species *Faecalibacterium prausnitzii*, one of the most dominant commensal microbes in the human gut.^9,10^ Importantly, *F. prausnitzii* is one of the many commensals that has been selectively modulated in order to restore the microbiota composition and improve intestinal health.^10^ In this respect, *F. prausnitzii* is an attractive target to restore intestinal homeostasis, since it can metabolize a variety of dietary components, including dietary fibers.^11^

Most dietary carbohydrates are efficiently degraded in the small intestine to di- and monosaccharides, which are subsequently absorbed in the small intestine, with very little spill-over into the colon.^12^ In contrast, dietary fibers reach the colon without being processed in the proximal GI tract and serve as essential carbon and energy sources for gut bacteria.^13^ Non-digestible food components that have the ability to selectively promote growth and metabolism of commensal gut bacteria are defined as prebiotics.^14^ Relevant examples include inulin-type fructans (ITFs), pectins and resistant starch. Inulin consists of oligo- or polymers of D-fructose molecules (fructans) with one D-glucose molecule at the terminus of each polysaccharide chain. A number of commensal bacteria, including bifidobacteria and faecalibacteria, are able to degrade the glycosidic bonds between fructose monomers in inulin and are therefore sensitive to this class of prebiotics.^15^

As a consequence of luminal content metabolism, *F. prausnitzii* is able to produce large amounts of short-chain fatty acids (SCFAs), especially butyrate, which are secreted into the intestinal lumen.^16^ Butyrate is specifically known for its anti-inflammatory and anti-carcinogenic properties, serving also as a preferred energy source for the intestinal epithelium.^17^ Additionally, butyrate contributes to immune system activation and enhances gut barrier function.^18–20^ However, besides the production of SCFAs, little is known about the metabolites generated by *F. prausnitzii* through prebiotic degradation, which may be beneficial for the human intestinal epithelium.^21,22^

The aim of this study was to dissect host-microbe cross-feeding (e.g., syntrophy) of metabolites generated from prebiotics making use of the “Human-oxygen Bacteria-anaerobic” (HoxBan) *in vitro* coculture system in order to investigate the molecular actions and benefits of prebiotics to intestinal epithelial health.

## Results

### Metabolism of prebiotics by F. prausnitzii decreases inflammatory markers in Caco-2 cells

**Figure 1.**
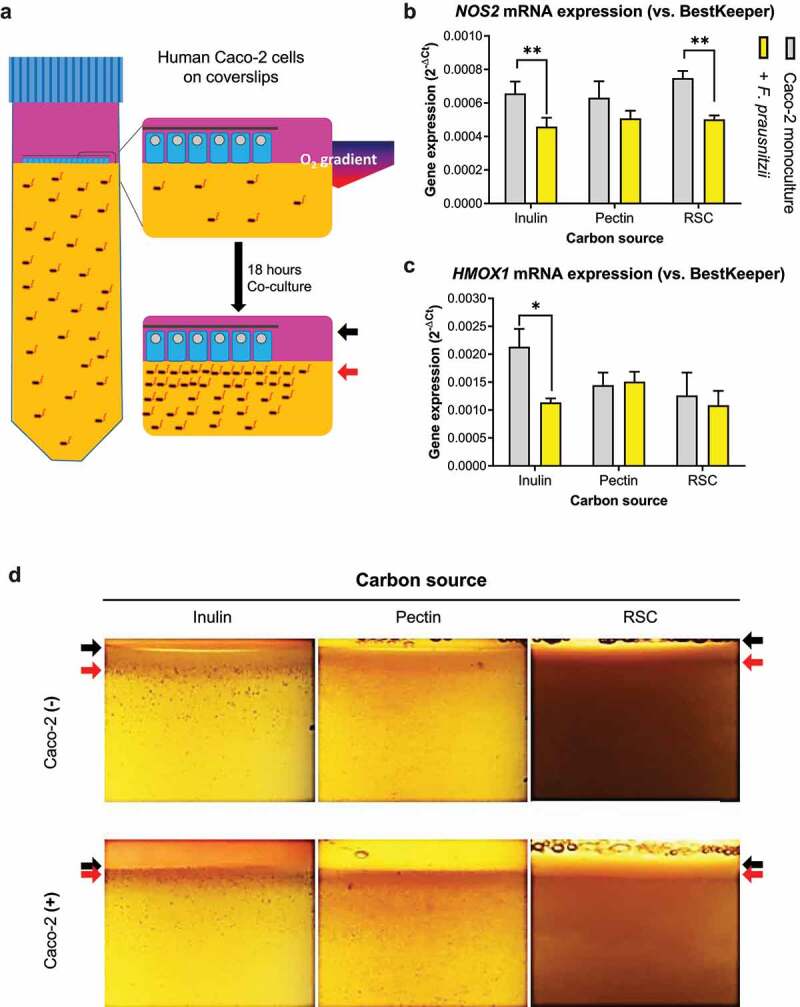
Metabolism of prebiotics by *F. prausnitzii* decreases inflammatory markers in Caco-2 cells. (a) Schematic representation of the HoxBan system, showing the oxic-anoxic interphase created between the Caco-2 monolayer (in human compartment, pink colored) and bacterial compartment (yellow color, representing the YCFA medium). The zoom schematically shows rim formation after 18-hour co-culture, where black arrows indicate the coverslip and red arrows indicate bacterial rim localization. (b-c) Effect of different prebiotic carbon sources on *F. prausnitzii*-regulated expression of *NOS2* (inflammation marker, **B**) and *HMOX1* (oxidative stress marker, **C**) in Caco-2 cells. Especially inulin-grown *F. prausnitzii* suppresses *NOS2* and *HMOX1* expression. (d) Prebiotic-grown *F. prausnitzii* in the absence and presence of Caco-2 cells. In the absence of Caco-2 cells (top panels), *F. prausnitzii* growth is enhanced (red arrow) in the upper part of the bacterial compartment forming a rim below the oxic-anoxic interphase (black arrow). In the presence of Caco-2 cells (bottom panels), *F. prausnitzii* growth is further enhanced (red arrow) and colonies appear closer to the oxic-anoxic interphase where the Caco-2 cells reside (black arrow). All experiments were performed with two biological replicates, each with an *N* = 3. *P < .05; **P < .01

Earlier, we reported that glucose-grown *F. prausnitzii* exerts anti-inflammatory and anti-oxidant effects on Caco-2 intestinal epithelial cells when cocultured in the HoxBan system.^23^ As glucose is not a prominent carbon and energy source for bacteria in the colon, we here aimed to fully replace glucose with prebiotics, e.g., inulin, pectin or resistant starch, in the bacterial compartment and analyze the effect on cocultured intestinal epithelial cells. Thus, Caco-2 intestinal epithelial cells were cultured in the HoxBan system with or without *F. prausnitzii* (the latter condition termed “monoculture”). Figure 1a shows a schematic representation of the HoxBan system. Inulin-grown *F. prausnitzii* significantly reduced mRNA levels of inflammatory (*NOS2*) and oxidative stress (*HMOX1*) markers in Caco-2 cells when compared to Caco-2 monocultures (Figure 1b,c), as observed earlier for glucose-grown *F. prausnitzii*.^23^ Similar trends were observed for pectin- or resistant starch-grown *F. prausnitzii*, but not as pronounced as for inulin (Figure 1b,c).

Bacterial growth in the HoxBan tube was assessed by visual inspection of colony formation close to the coverslip with the Caco-2 cell monolayer (Figure 1d). Both in the absence and presence of Caco-2 cells, *F. prausnitzii* growth is enhanced (forming a rim of colonies, red arrows in Figure 1d) close to the interphase between the bottom anaerobic (bacterial) compartment and the upper oxygenated (human cell) compartment (black arrows in Figure 1d). This was observed for all fibers, as also observed earlier for glucose-grown *F. prausnitzii* in the HoxBan system.^23^ Importantly, the distance between the *F. prausnitzii* rim and the coverslip in the human cell compartment was smaller in all conditions in the presence of Caco-2 cells (red arrows in bottom panels Figure 1d) when compared to empty coverslips (red arrows in top panels Figure 1d). Thus, Caco-2 cells promote growth of fiber-fed *F. prausnitzii* closer to the epithelial oxygen-anaerobic interphase.

### 1. Digestion of inulin and pectin by *F. prausnitzii* promotes Caco-2 viability even in the complete absence of glucose

**Figure 2.**
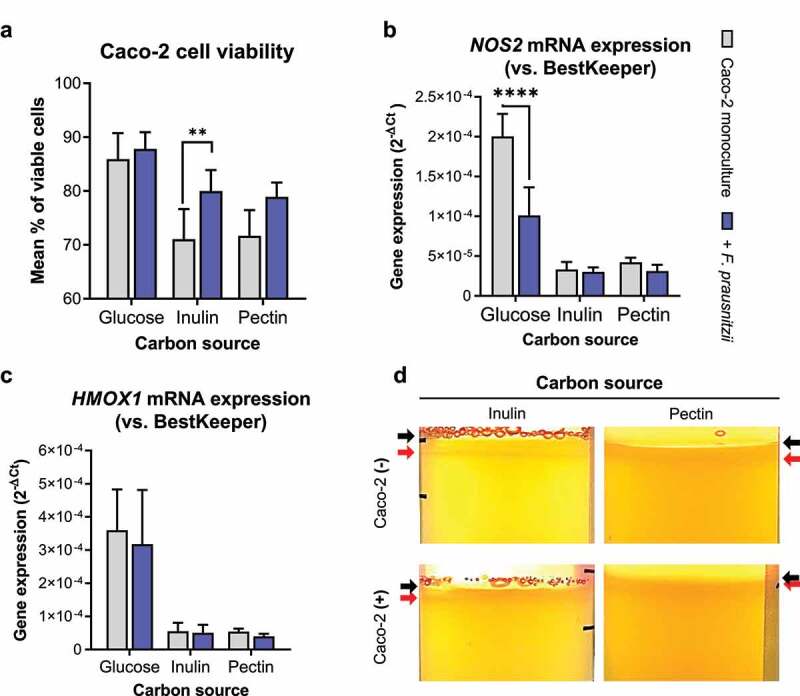
*F. prausnitzii* grown on inulin improves Caco-2 cell viability when cocultured in the complete absence of glucose. (a) Caco-2 cell viability after 18 h coculture without (gray bars) or with (blue bars) *F. prausnitzii* in the HoxBan system containing glucose, inulin or pectin as sole carbon and energy source. (b, c) Corresponding mRNA levels of *NOS2* (b) and *HMOX1* (c) in the *F. prausnitzii*-Caco-2 coculture shown in **A**. Basal levels of *NOS2* and *HMOX1* are reduced in Caco-2-*F. prausnitzii* cocultures on inulin and pectin when compared to glucose, with no significant additional effect of the presence of *F. prausnitzii*. (d) In the complete absence of glucose, inulin- or pectin-grown *F. prausnitzii* forms a growth rim in the upper part of the bacterial compartment (red arrows), which is closer to the coverslip (black arrows) containing Caco-2 cells (bottom panels) compared to empty coverslips (top panels). All experiments were performed with two biological replicates, each with an *N* = 3 (inulin) and 2 (pectin). **P < .01; ****P < .0001

The culture medium for Caco-2 cells in the HoxBan system (e.g., DMEM) contains 4.52 g/L (25 mM) glucose, which may partly penetrate into the bacterial compartment and also support growth of *F. prausnitzii*. Indeed, omitting glucose completely from the bacterial compartment in the HoxBan system, while maintaining normal glucose levels in the human (upper) compartment, still led to bacterial growth and a non-significant decrease in *NOS2* and *HMOX1* mRNA levels in Caco-2 cells (**Supplementary Figure S1A-D**). Aiming to determine whether fiber-fed *F. prausnitzii* can provide sufficient essential metabolites for Caco-2 cells, glucose was excluded completely from both (human cell and bacterial) compartments of the HoxBan system, and only inulin or pectin was provided in the bottom (bacterial) compartment (Figure 2). Caco-2 cells show 85.9% cell viability after 16 h monoculture in the HoxBan system with glucose in the lower (bacterial) compartment, which drops to 71.1% and 71.7% when glucose is replaced by inulin or pectin, respectively. However, Caco-2 cell viability significantly increased (to 80.0%) when *F. prausnitzii* was cocultured on inulin (Figure 2a). Similar trends in promoting Caco-2 cell viability were observed for pectin- and glucose-grown *F. prausnitzii* (79.9% and 87.8%, respectively). When compared to glucose, basal mRNA levels of *NOS2* and *HMOX1* were strongly reduced in Caco-2 cells monocultured on inulin or pectin, which were not significantly changed in the presence of *F. prausnitzii* (Figure 2b-c). Again, bacterial growth was observed closer to the coverslip with Caco-2 cells when compared to empty coverslips (Figure 2d), similar as observed for conditions where glucose was present in the human (upper) compartment (Figure 1d).

### Inulin-grown *F. prausnitzii* produces excess fructose, which is a carbon source for Caco-2 cells.

**Figure 3.**
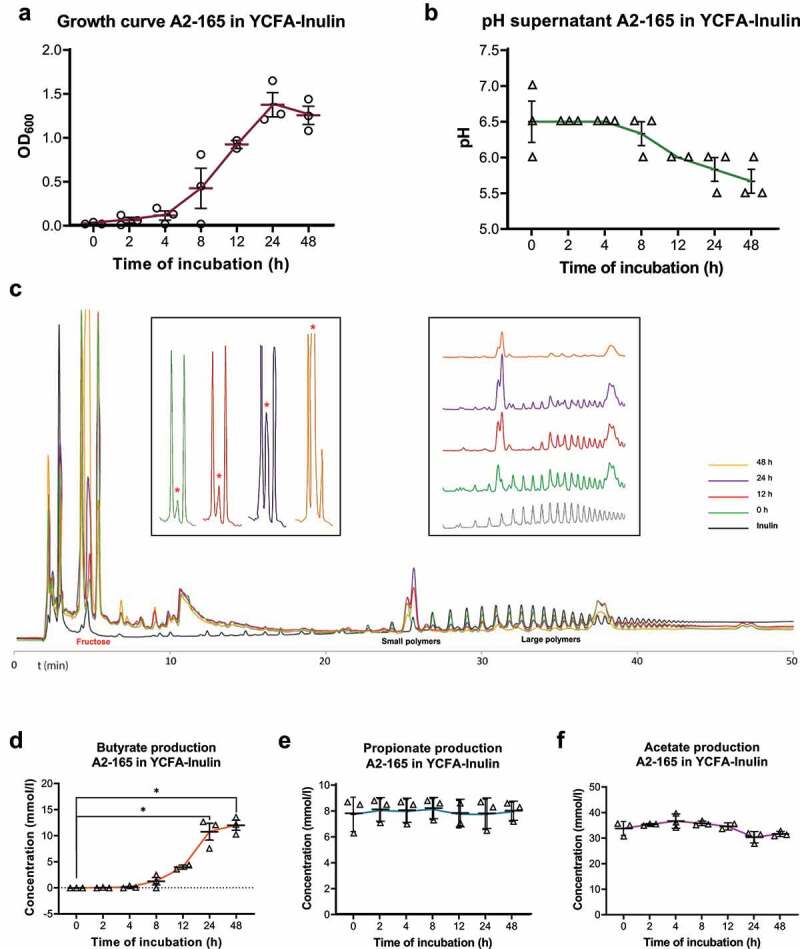
*F. prausnitzii* produces excess fructose and butyrate from inulin. *F. prausnitzii* was grown in bacterial broth containing inulin as sole sugar source and analyzed for (a) bacterial growth, (b) medium acidification, (c) inulin metabolism and fructose production, as well as the production of the short-chain fatty acids (SCFAs) butyrate (d), propionate (e) and acetate (f). Time-dependent growth of *F. prausnitzii* is associated with medium acidification, decrease in inulin (large polymers in **C**) and increase in fructose (asterisks in inset in **C**) and butyrate (**D**). Note that fructose levels increase particularly when *F. prausnitzii* is in the stationary growth phase (24–48 h), while butyrate levels increase most during the exponential growth phase (12–24 h). Propionate and acetate are endogenous components of the bacterial broth (at 8 and 30 mM, respectively) and their concentrations do not significantly change during *F. prausnitzii* growth on inulin. All experiments were performed with two biological replicates, each with an *N* = 3. *P < .05

We next aimed to determine which metabolite(s) produced from inulin by *F. prausnitzii* could support Caco-2 cell growth and viability. First, *F. prausnitzii* was cultured in anaerobic batch cultures on inulin as sole carbon and energy source (Figure 3). Exponential growth was observed during the first 24 h, after which *F. prausnitzii* entered a stationary growth phase (Figure 3a). Bacterial growth was accompanied by a drop in pH of the medium from 6.5 to 5.5 (Figure 3b), indicating high metabolic activity of *F. prausnitzii* in both growth phases. Qualitative analysis of inulin and its breakdown products by High pH Anion Exchange Chromatography (HPAEC) revealed that medium-sized polymers (elute at 25–35 min) are first converted to smaller fructose polymers (elute around 25 min), which peak after 24 h *F. prausnitzii* growth (Figure 3c, right inset). The larger fructose polymers (elute at 35–40 min) are subsequently consumed when *F. prausnitzii* enters the stationary growth phase (between 24 and 48 h). Interestingly, the concentration of fructose monomers in the medium increases in a time-dependent manner, especially accumulating in the stationary growth phase of *F. prausnitzii* between 24 and 48 h (Figure 3c, fructose elutes at 5 min, see also red asterisks in left inset). Butyrate levels also increased in a time-dependent manner in the medium up to 10 mM after 24 h of *F. prausnitzii* culture. In contrast to fructose levels, butyrate levels did not significantly increase further during the stationary phase (Figure 3d). Propionate (~8 mM) and acetate (~30 mM) are standard components of the YFCA culture medium for *F. prausnitzii* and remained relatively stable throughout the exponential and stationary growth phases, although after 24 hours a drop was seen in the acetate concentration, due to consumption together with fructose (Figure 3e,f).

### Fructose, and not butyrate, supports proliferation of Caco-2 cells

**Figure 4.**
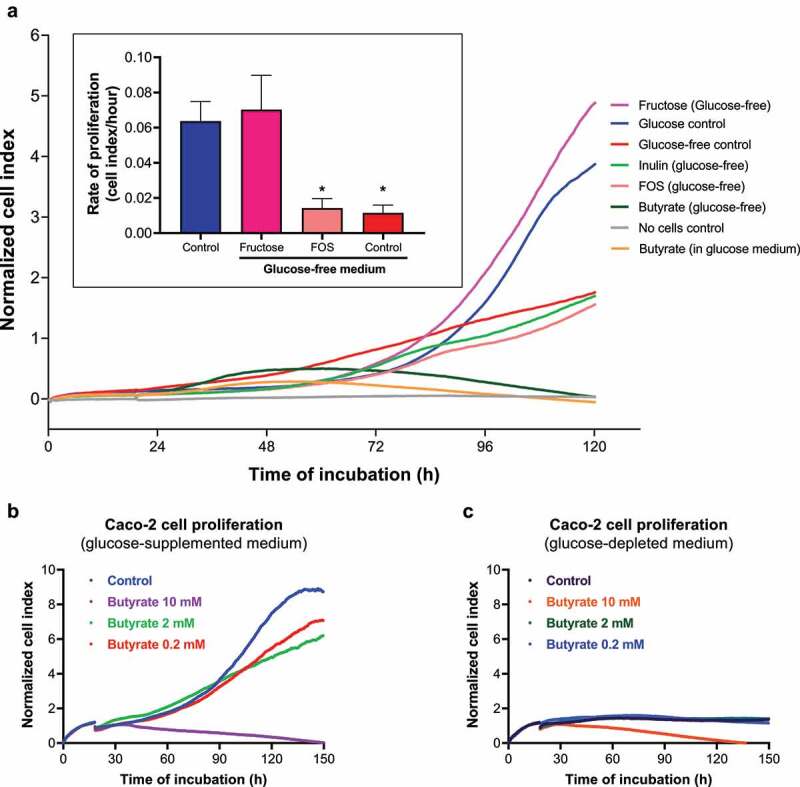
Caco-2 cells grow on fructose, but not on butyrate or inulin. (a) Caco-2 cells were cultured in a real-time cell analyzer (RTCA, xCELLigence) to monitor cell growth on different carbon/energy sources, e.g., glucose (as positive control, dark blue line), fructose (pink line), butyrate (dark green), inulin (light green) or fructose-oligosaccharide (FOS, rose line) and compared to cell cultured in the absence of a carbon and energy source (bright red line). The inset in **A** shows the maximum growth rate (in Δcell index/h) of Caco-2 cells on glucose (blue bar), fructose (purple bar) and FOS (rose bar) when compared to the no added carbon source (red bar). Caco-2 cells grow at a similar pace on glucose and fructose. In contrast, butyrate causes only an initial small increase in cell index (green line between 24–48 h), after which the cell index goes down, suggesting cell death. In fact, butyrate blocks growth of Caco-2 on glucose (Orange line). Butyrate decreases Caco-2 cell proliferation in a dose-dependent manner both in (b) glucose-supplemented and (c) glucose-depleted media. All experiments were performed with two biological replicates, each with an *N* = 3 (glucose and inulin) and 2 (pectin). *P < .05

Next, we analyzed whether the metabolites produced by inulin-fed *F. prausnitzii*, e.g., fructo-oligosaccharides (FOS), fructose and/or butyrate, as well as inulin itself can support growth of Caco-2 cells. Caco-2 cells were cultured for 5 days in DMEM medium without carbon source or supplemented with glucose (= standard medium as reference), fructose, FOS, inulin or butyrate (Figure 4a). Moreover, to tested the effect of butyrate on cell proliferation in the presence or absence of glucose (Figure 4b,c). Caco-2 cell proliferation was monitored in real-time using the xCELLigence system. Cell index (as a measure of cell proliferation) of Caco-2 cells grown on glucose increased exponentially after an initial lag phase and entered the stationary phase after approximately 110 h culture (Figure 4a, blue line). A similar growth profile was observed for Caco-2 cells grown on fructose (Figure 4a, purple line). In contrast, the cell index of Caco-2 cells grown in DMEM supplemented with FOS (Figure 4a, rose line) or inulin (Figure 4a, light green line) was similar to Caco-2 cells grown in a glucose-free medium (Figure 4a, red line). The maximum rate of cell proliferation (in cell index/hour; inset on Figure 4) was similar for Caco-2 cells grown on glucose and fructose, while FOS did not induce cell proliferation above cells in the glucose-free conditions.

Remarkably, low concentrations of butyrate (2 mM) in combination with glucose (Figure 4b), only slightly and transiently promoted Caco-2 cell proliferation in the early lag phase (up to 48 h), after which the cell index actually decreased in a dose-dependent manner, suggesting a cytotoxic effect on Caco-2 cells. Importantly, butyrate as a sole carbon source (Figure 4c) did not promote Caco-2 cell proliferation at any of the tested concentrations (0.2, 2 or 10 mM), with growth curves resembling the one of glucose-depleted control. Together, these data indicate that butyrate (in the presence or absence of glucose) is not a primary growth substrate for Caco-2 cells.

### Inulin-grown *F. prausnitzii* enhances expression of the fructose transporter *SCL2A5* in Caco-2 cells.

**Figure 5.**
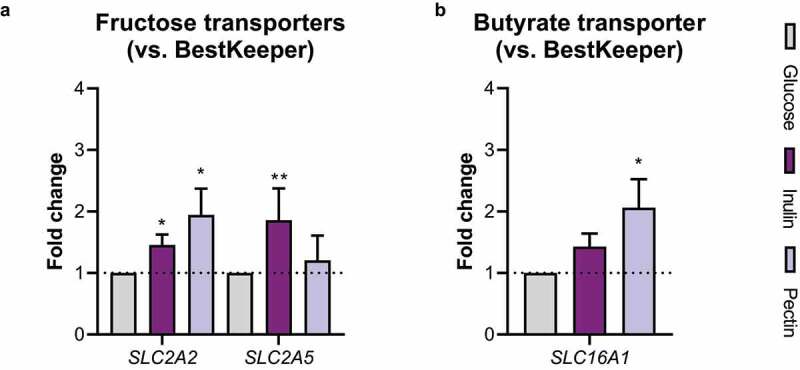
Inulin-grown *F. prausnitzii* increases expression of fructose transporters in Caco-2 cells. Caco-2 cells were cocultured with *F. prausnitzii* in the HoxBan system containing either glucose, inulin or pectin as sole carbon and energy source and analyzed for gene expression of **A**) the fructose transporters *SLC2A2* and *SLC2A5* (encoding GLUT2 and GLUT5 respectively) and **B**) the butyrate transporter *SLC16A1* (encoding MCT1). Inulin-grown *F. prausnitzii* significantly induced gene expression of both fructose transporters. All experiments were performed with two biological replicates, each with an *N* = 3. *P < .05; **P < .01

Fructose regulates its own cellular absorption by increasing transcription of, among others, *SCL2A2* and *SLC2A5*, genes encoding the fructose transporters GLUT2 and GLUT5, respectively.^24,25^ *SLC2A2* and *SLC5A2* mRNA levels were indeed significantly enhanced in Caco-2-*F. prausnitzii* HoxBan cocultures when inulin was the only carbon source present, when compared to glucose as sole carbon source (Figure 5a). Such significant *SLC2A5* upregulation was not observed when pectin was provided as sole carbon source, although upregulation of *SLC2A2* was significant in these conditions. Furthermore, significant upregulation of the butyrate transporter MCT1 (encoded by the *SLC16A1* gene) was **observed** in pectin-HoxBan cocultures, compared to the glucose condition (Figure 5b). Gene expression levels of the other butyrate transporter SMCT-1 (encoded by the gene *SLC5A8*) were undetectable. Taken together with previous results, this shows that *F. prausnitzii* produces fructose monomers from inulin and that Caco-2 cells use the fructose as carbon and energy source.

### Fecal concentrations of fructose and glucose associate with levels of *F. prausnitzii* in a population-based cohort

**Figure 6.**
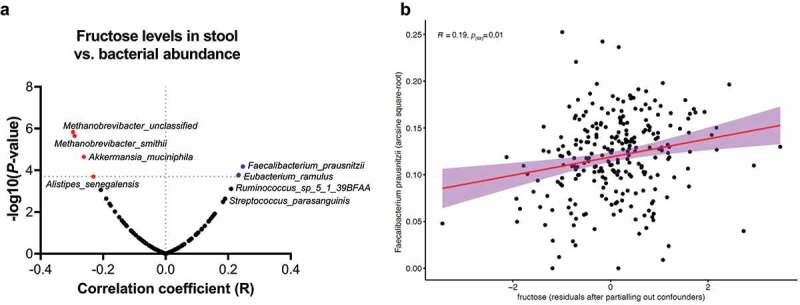
Fecal fructose levels positively correlate with *F. prausnitzii* abundance. (a) Volcano plot showing bacterial species whose relative abundance correlates significantly with fecal fructose levels in a population cohort (n = 255). Transformed *F. prausnitzii* levels most strongly correlate positively with fecal fructose levels (*R* = 0.19, *p_(fdr)_ *=^ ^0.01; **B**), while *A. muciniphila* is amongst species that show a significant negative correlation with fecal fructose

Concentrations of monosaccharides, e.g. glucose and fructose, are typically assumed to be low in the colon.^12,26^ Still, the above observation of bacterial fructose production from fibers prompted us to specifically analyze fructose and glucose levels in an unbiased metabolome analysis of 255 fecal samples from a population cohort, and analyze their putative correlation with the taxonomical composition of the microbiota (Figure 6). In order to account for potential confounders, we first investigated the relation between host- and dietary factors and fecal glucose and fructose levels. In total, we found 9 factors associated with the levels of the metabolites (**Suppl. Table S3**). These factors were then used as a covariate in the linear regression model to determine the residual-corrected association between the relative abundance o *F. prausnitzii* and glucose or fructose levels. Fructose and glucose were readily detected in stool samples of the study population. Remarkably, linear regression analysis (represented in a volcano plot; Figure 6a) of 112 bacterial species reveals that *F. prausnitzii* abundance showed the most significant positive correlation with stool fructose levels (*R* = 0.19, *P_(fdr)_*= 0.01 Figure 6b). Abundance of only one other species, *Eubacterium ramulus*, also correlated positively with fructose levels in stool, while 4 species correlated negatively to fructose levels in the stool (e.g., *Methanobrevibacter* sp., *Methanobrevibacter smithii, Akkermansia muciniphila* and *Alistipes senegalensis*). *F. prausnitzii* abundance also positively correlated with glucose levels in stool (*R* = 0.28 and *P_(fdr)_*= 4.3 × 10^−6^; **Supplementary Figure S3)**, though the highest significance for a positive association with glucose stool levels was observed for *Eubacterium rectale*.

## Discussion

In this study, we show that the dominant colonic commensal microbe *F. prausnitzii* produces excess butyrate and fructose when metabolizing the prebiotic inulin. Moreover, fructose was readily detected in 255 fecal samples of a large population cohort and positively correlated with *F. prausnitzii* abundance. Caco-2 intestinal epithelial cells use fructose as a carbon and energy source, whereas they do not grow on butyrate. When cocultured *in vitro* in the HoxBan system with inulin as sole carbon and energy source, the strict anaerobe *F. prausnitzii* enhances Caco-2 cell viability, induces expression of fructose and butyrate transporters and suppresses inflammatory and oxidative stress signaling in the human intestinal epithelial cells. Thus, bacterial breakdown of fibers/prebiotics to simple sugars/monosaccharides in the colon provides a constant fuel for epithelial cell proliferation while suppressing inflammation and oxidative stress. These data call for a reevaluation of the relative contribution of different bacterial metabolites generated from fibers/prebiotics, including butyrate and fructose, to gut health.

The prevailing concept of the action of prebiotics is that colonic bacteria feed on them thereby producing high amounts of short-chain fatty acids (SCFA), including butyrate that is a preferred energy source for colonocytes. Additionally, butyrate exerts various anti-inflammatory actions to promote gut health. Indeed, seminal work in the early 1980s showed that the rate of oxygen consumption is higher when primary human colonocytes are treated for 60 min with butyrate, when compared to glucose.^27,28^ Subsequent studies have supported these observations, for instance by using human intestinal epithelial cell lines (Caco-2 and T84). Treating these cells with butyrate in the presence of glucose increased oxygen consumption and saturation by increasing mitochondrial activity and activation of the oxygen-sensitive pathway hypoxia-inducible factor 1α (HIF1α).^29,30^ Together, these data indeed show that butyrate is an efficient energy source for colonocytes. However, exposure times are typically between minutes and several hours and do not establish butyrate as a carbon source for macromolecule biosynthesis to promote cell growth and proliferation. In human cells, butyrate is oxidized by mitochondria (β-oxidation) and yields acetyl-CoA as an end-product, which efficiently fuels ATP production.^31^ Human cells cannot convert acetyl-CoA to glucose, the universal carbon source for macromolecule biosynthesis in mammalian cells, consequently excluding biosynthetic functions of butyrate in intestinal epithelial cells.^32,33^ In fact, *in vitro* treatment of colonic crypts, crypt derived organoids and noncancerous cell lines (NCM460 and FHC) shows that butyrate reduces cell proliferation and induced cellular senescence.^34–36^ Fructose, on the other hand, is efficiently converted to glucose in the intestinal epithelium.^37,38^

Inulin is a fructose polymer with a glucose molecule at the terminus of each chain. Degradation of inulin and FOS by commensal gut bacteria has been extensively studied. Some butyrate-producing bacteria, like *F. prausnitzii*, can grow on inulin as primary carbon and energy source,^39^ others depend on cross-feeding of smaller metabolites generated by for instance bifidobacterial.^40^ Inulin-supplemented *in vitro* coculture of *Lactobacillus acidophilus* (IBB 801), *Lactobacillus paracasei* (8700:2) and *Bifidobacterium longum* (LMG 11047) resulted in release of free fructose to the media, together with lactate and acetate.^41^ Furthermore, *in vitro* fermentations of inulin by diluted stool samples showed a peak of fructose production after 8 h of incubation, which coincided with acidification of the media and decreased at 24 h of culture (**Suppl. Figure S2A** and **B**). These experiments demonstrated that the cross-feeding between different bacterial species still results in accumulation of fructose that is made available to intestinal epithelial cells. In our work, we found that fructose production was particularly increased when inulin-grown *F. prausnitzii* entered the stationary growth phase. In contrast, butyrate levels did not further increase then. A recent study made a similar observation^39^ with another strain of *F. prausnitzii* (DSM 17677®). These results imply that butyrate production from inulin requires (exponential) bacterial growth, while this is not the case for fructose production. *F. prausnitzii* likely secretes enzymes that breakdown the inulin polymers, enzymes that continue doing so when bacterial growth ceases. Upregulation of β-fructofuranosidase, as well as coupled ABC transporter protein, have been described in *Roseburia intestinalis* (a Firmicutes gut commensal and butyrate producer, as *F. prausnitzii*) during growth on inulin.^42,43^ Nevertheless, this has not yet described for *F. prausnitzii* grown on inulin-type fructans. The fecal fructose levels are likely a result of such bacterial fiber fermentation, since dietary monosaccharides are effectively absorbed in the small intestine. Interestingly, of all bacteria detected, *F. prausnitzii* abundance showed the strongest positive correlation to fructose levels in fecal samples, which may suggest that *F. prausnitzii* is a contributing factor in producing fructose, or monosaccharides in general, in the colon from dietary fibers and thereby cross-feeding to the colonic epithelium.

The presence of fructose in feces also implies that it is always available as a carbon and energy source for the human colonic epithelium and not completely used by other bacteria. Cross-feeding of metabolites produced by inulin-grown *F. prausnitzii* to Caco-2 intestinal epithelial cells was reproduced *in vitro* using the HoxBan system, which allows coculturing of strict anaerobes like *F. prausnitzii* and oxygen-requiring human intestinal epithelial cells.^23^ Caco-2 cell viability was improved when cocultured with inulin-grown *F. prausnitzii*, when compared to conditions with bacteria. We were not able to detect significant amounts of fructose in the medium after coculture in the HoxBan system, but this is likely a result of efficient absorption of the produced fructose by the Caco-2 cells. Gene expression of *SLC2A2* and *SLC2A5* (encoding the fructose transporters GLUT2 and GLUT5) is induced by fructose,^12^ which was also observed in Caco-2 cells cocultured with inulin-grown *F. prausnitzii*. GLUT5 is the main fructose transporter situated at the apical membrane of intestinal epithelial cells, and its deletion completely abrogates transepithelial fructose transport, highlighting its physiological importance.^44,45^ In contrast, GLUT2, situated at the basolateral membrane, is not specifically upregulated by high luminal fructose contents, as it serves multiple substrates (also glucose and galactose) and its deletion only modestly impairs fructose absorption.^46^ In line with this, the longstanding notion that GLUT2 is the main apical fructose transporter has been excessively scrutinized.^24^ Our data also indicated that GLUT5 is most sensitive to inulin with an approximately 2-fold increase in expression in inulin-grown Caco-2/*F. prausnitzii* cocultures. Interestingly, inulin-grown *F. prausnitzii* also suppressed expression of *NOS2* and *HMOX1*, as sensitive markers of inflammation and oxidative stress. Lastly, we observed that fructose and butyrate transporters are also upregulated in Caco-2 cells in coculture with pectin-grown *F. prausnitzii*. However, further research is needed to understand the cross-feeding from pectin metabolites to intestinal epithelial cells.

Various clinical trials have been conducted to increase the abundance of beneficial bacteria like *F. prausnitzii* and *A. muciniphila* by dietary factors (reviewed by Verhoog et al. (2019)). Most of these studies reported that diets enriched for fructo-oligosaccharides (FOS), inulin or raffinose enhance *F. prausnitzii* levels that, based on our data, would in turn provide multiple carbon and energy sources to the colonic epithelium and improve gut health.

In conclusion, this study shows that fermentation of inulin by *F. prausnitzii* produces excess fructose, a carbon and energy source that is readily available for the human colonic epithelium. As *F. prausnitzii* levels are decreased in various metabolic and digestive diseases, including IBD,^47–52^ which are also characterized by a leaky gut, promoting *F. prausnitzii* abundance and/or metabolism by administration of prebiotics like inulin may provide the highly required carbon and energy sources to support effective epithelial regeneration.

## Materials & methods

### RESOURCE AVAILABILITY

#### Lead contact

Further information and requests for resources and reagents should be directed to and will be fulfilled by the Lead Contact, Prof. Klaas Nico Faber k.n.faber@umcg.nl.

#### Materials availability

TaqMan primers and probes for RT-qPCR generated in this study are described in Table 1. *F. prausnitzii* (strain A2-165) was kindly provided by S. Duncan and H. Flint (Rowett, UK).

#### Data and Code Availability

The raw metagenomics sequencing data used for this study are available from the European Genome-phenome Archive data repository: 1000IBD cohort (https://www.ebi.ac.uk/ega/datasets/EGAD00001004194) and LifeLines DEEP cohort (https://www.ebi.ac.uk/ega/datasets/EGAD00001001991). Due to participant confidentiality, the datasets are available upon reasonable request to the University Medical Center of Groningen (UMCG) and LifeLines, respectively. The metabolomics data supporting the current study have not yet been deposited in a public repository because of participant confidentiality, but are available from the corresponding author on request.

## Experimental model and subjects details

### Cell lines and bacterial strains

Human epithelial colon adenocarcinoma cells (Caco-2, male, ATCC®, HTB-37^TM^) were used as representative of intestinal epithelial cells. Cells were incubated at 37°C and 5.0% CO_2_. As previously described in Sadaghian Sadabad *et al* (2015), Caco-2 cells were cultured in Glutamax™ Dulbecco’s Modified Eagle Medium (DMEM, ThermoFisher Scientific Inc) with 25 mM glucose and supplemented with 10% fetal calf serum (FCS, Invitrogen), 1% non-essential amino acids (NEAA, Gibco) and 1% PSF antibiotic cocktail (penicillin (10 U/ml), streptomycin sulfate (100 μg/ml) and fungi zone; Lonza). For use in the HoxBan system, cells were seeded on coverslips until 70–80% confluence in a 12-well plate (4 cm^2^/well), for 24 hours. About 1 hour before transferring the coverslip-attached Caco-2 cells to the coculture system, the medium in each well was discarded and replaced by PSF-free DMEM medium.

*F. prausnitzii* (A2-165) was cultured anaerobically at 37°C starting from −80°C glycerol stocks (20% glycerol in YCFAG medium) in YCFAG medium, which contains 2.5 g/l yeast extract, 10.0 g/l casitone, fatty acids (9 mM propionate, 1 mM isobutyrate, 1 mM isovalerate and 1 mM valerate), 33 mM sodium acetate and 4.52 g/l (20 mM) glucose (detailed medium composition can be found in Sadaghian Sadabad *et al* (2015)). In specific experiments, glucose was omitted from YCFAG medium or replaced by 4.52 g/l of specified fibers, e.g., inulin (I; Raftiline® HP, CAS number 9005–80-5, ORAFTI S.A.), apple pectin (P; catalog number 93854 Sigma-Aldrich) or resistant starch (RSC; catalog number S4126, Sigma-Aldrich). Media is further described on **Suppl. Table S1**. *F. prausnitzii* was inoculated from frozen stocks at a dilution of 1:1,000 in 5 ml YCFAG, -I, -P or -RSC for 14–16 hours until an optical density (at 600 nm) of 0.8.

### Human studies

Population-based fecal metagenomics and fecal metabolomics data were derived from the LifeLines-DEEP cohort available at the time of conducting this research, a Dutch general population-based cohort study consisting of individuals from the Northern part of the Netherlands.^53^ Metagenomics and untargeted metabolomics data were available from a subset of this cohort (n = 255, 114 males (44.7%, average age 46.8 (S.D. 12.8), average BMI 25.17 (S.D. 3.8)) for which microbiome data was also available and after excluding subjects with inflammatory bowel diseases. Participants were requested to collect and freeze fecal samples at home. Samples were then picked up and transported on dry ice and stored at −80°C until further analysis. All participants provided written informed consent prior to sample collection (IRB ref. M12.113965).

## Methods details

### HoxBan coculture system

As described by Sadaghian Sadabad *et al*. (2015), anaerobically-grown *F. prausnitzii* was inoculated in YCFAG liquid broth, from which 1 ml was used to inoculate 1 liter of autoclaved agar-based (1% agar) YCFAG, -I, -P or -RSC (pH = 6.5) and cooled-down to approximately 40°C. For each 50 ml Falcon test tube, 40 ml of this mixture was added in an anaerobic cabinet and transferred to a laminar flow cabinet and opened at ambient air. 10 ml of 37°C PSF-free DMEM was added to each tube. Next, the Caco-2 cells grown on coverslips were laid upside-down on the top of the bacteria-containing agar medium. The screw caps of the Falcon tubes were kept loosely tightened, to allow oxygen entry into the system for the Caco-2 cells. Coculture took place for 18 hours at 37°C and 5% CO_2_, after which coverslips were collected and processed for downstream analyses.

## RNA isolation and gene expression quantification

RNA was isolated from Caco-2 cells using TRIzol (Sigma-Aldrich) according to the manufacturer’s protocol (Thermo-Scientific), followed by quantification of RNA using a NanoDrop 2000 c spectrophotometer. To synthesize complementary DNA (cDNA), 2.5 ng of RNA was added to a final volume of 50 μl, 10% reaction RT Buffer (final concentration 50 mM Tris-HCl, 50 mM KCl, 3 mM MgCl_2_, 5 mM DTT), 10% dNTP mix (final concentration 1 mM dATP, dGTP, dTTP, dCTP, Sigma-Aldrich), 2% random primers (0.01 μg/μl; Sigma-Aldrich), 2% M-MLV RT (100 U; Invitrogen) and 1.5% RNAse OUT (30 U; Invitrogen). cDNA synthesis was performed on a thermal cycler (Bio-Rad T100) for 10 minutes at 25°C followed by 60 minutes at 37°C and 5 minutes at 95°C. Prior to quantitative real-time PCR, cDNA solution for each sample was diluted 20-fold in RNAse-free water.

Gene expression of inducible nitric oxide synthase (*NOS2*), heme oxygenase 1 (*HMOX1*), interleukin-1 beta (*IL1B*), glucose transporter 2 (GLUT2) (*SLC2A2)* and glucose transporter 5 (GLUT5) (*SLC2A5)* were measured by TaqMan-based quantitative Real-Time PCR (RT-PCR). BestKeeper Index analysis of the housekeeping genes *18S, GAPDH, RPII* and *ACTB* was used for normalization of gene expression levels of genes of interest (software available at https://www.gene-quantification.de/bestkeeper.html). The sequence and description of the probes and primers used in this study are presented in **Supplementary Table S2**. Each sample was prepared in duplicates, in a reaction mix (final volume 20 μl) containing 0.2 μM fluorescent probes, 0.936 μM forward and reverse primers, 10 μl qPCR reaction buffer (Eurogenic), 4.48 μl RNAase-free water and 4 μl cDNA. Amplification was performed after 10 minutes of heating at 95°C followed by a 40-times repeated cycle, consisting of 15 seconds at 95°C and 1 min at 60°C, using a StepOnePlus (AB, Applied Biosystems) PCR system.

## Cell viability assay

Viability of Caco-2 cells was assessed by Trypan blue staining. Trypan blue solution (0.2%, Sigma-Aldrich) was added to the coverslip-attached Caco-2 cells (500 μl/well) for 1 minute, after which Caco-2 cells were fixed for 10 minutes at RT using 4% paraformaldehyde (PFA; Sigma-Aldrich) in phosphate-buffered saline solution (PBS; catalog 10010023, Gibco®, ThermoFisher Scientific). Adherent cells were rinsed 4 times with PBS and fixed on top of glass plates using mounting medium (Thermo-Fisher Scientific). Quantification of cell viability was performed by manually counting Trypan blue-positive and – negative cells using light microscopy (at 200x magnification). Cell viability was expressed as the mean percentage of viable cells per field.

### Proliferation assay

The xCELLigence® RTCA-DP system was used to assess Caco-2 cell proliferation, by monitoring electrical resistance buildup on well surface. 2,000 cells were seeded in an xCELLigence® 16-well and attachment was allowed overnight on standard DMEM medium, in a standard CO_2_ cell culture incubator. After the attachment phase, medium was replaced by either new standard or glucose-depleted DMEM medium with different carbon-sources in duplicate. Glucose, fructose, fructo-oligosaccharides (FOS) and inulin were added to a final concentration of 4.52 g/l, which is equal to the standard sugar concentration in the standard YCFAG bacterial agar in the HoxBan coculture system. Butyrate (Sigma-Aldrich) was added to concentrations ranging from 0.2 to 10 mM. The 16-well plate was placed in the xCELLigence system and cell index was measured every 15 min for 5 days, medium and conditions being refreshed every 2 days. Rate of cell proliferation (expressed as cell index/hour) was assessed by calculating the slope of growth curve at exponential phase for each treatment.

## *Inulin breakdown by* F. prausnitzii

From an overnight culture of *F. prausnitzii* in YCFAG (described above), 50 μl was inoculated in 5 ml YCFAI medium and statically incubated for 18 hours under anaerobic conditions. The resultant bacterial suspension was again diluted (1:100) in 50 ml YCFAI, and kept in anaerobic conditions at 37°C. Aliquots of 1 ml (in duplicates) were taken from this inoculum at indicated time points (0, 2, 4, 8, 12, 24 and 48 hours) and pH measured, followed by centrifugation (13,000 × *g* for 5 min) to remove bacteria. The supernatant was collected and stored at −20°C prior to fructose or SCFAs analysis by high-performance liquid chromatography (HPLC).

## Carbohydrate semi-quantitative analysis

1.0 ml supernatant aliquots (as described above) were transferred to a 1.5 ml tube and centrifuged at 13,000 × *g* at RT, the supernatants were diluted 1:5 in MilliQ water for analysis. Samples were analyzed by High pH Anion Exchange Chromatography (HPAEC) on an ICS300 system at the University of Groningen, Dept. Microbial Physiology, eluted with a 55 min linear gradient of NaOAc (30–600 mM) in 100 mM NaOH. Carbohydrates were detected with a Pulsed Amperometric Detector, using a quadruple potential waveform.^54^ A qualitative standard of equimolar glucose (G), fructose (F), sucrose (GF), kestose (GF_2_) and nystose (GF_3_) (0.1 mM) was used to identify FOS peaks. Confirmation of fructose and fructo-oligosaccharide peaks in samples was achieved by spiking 500 μl supernatant sample with 100 μl with 0.28 mM fructose or 100 μl 1 mg/mL small polymer inulin (Sensus, Roosendaal, The Netherlands).

## Short-chain fatty acid (SCFA) analysis

SCFA in bacterial supernatants were quantified as described by Moreau et al (2003) with minor modifications. Supernatant samples were thawed on ice and 85 μl was diluted in 415 μl MilliQ water. A 7-point calibration curve in Milli-Q was stored in aliquots at −80 °C with final concentrations between 0.0 and 8.0 mM for acetate and between 0.0 to 4.0 mM for propionate and butyrate. 100 μl internal standard solution (1 mg/ml 2-ethylbutyrate in Milli-Q), 20 μl 20% (w/v) sulphosalicylic acid solution and two drops 37% HCl (approximately 100 μl) were added to the 500 µl diluted supernatant samples and calibration samples. The samples were centrifuged at 16,100 × *g* for 20 min at 4°C, and the supernatant was transferred to a glass tube containing a spatula tip of sodium chloride (approximately 30 mg). 2 ml of diethyl ether was added and the sample was vortexed for 10 min at RT and centrifuged at 3,000 × *g* for 10 min at 4°C. From the clear upper layer, a 500 μl aliquot was taken and transferred to a glass GC-vial. Subsequently, 50 μl of MBTSTFA + 1% TBDMCS (N-methyl-N-(tert-butyldimethylsilyl)trifluoroacetamide + 1% tertbutyldimetheylchlorosilane) was added and left to derivatize overnight at room temperature. 3 μl derivatized sample was injected into the GC-MS (7890A GC System and 5975 C inert XI EI/CI MSD with an EI inert 350 source, Agilent Technologies, Santa Clara, USA). Analysis was carried out in a split mode with an inlet split ratio of 20:1. Samples were analyzed in SIM acquisition mode; acetate at m/z 117, propionate at m/z 131, butyrate at m/z 145 and 2-ethylbutyrate at m/z 175. Injector, source and quadrupole temperatures were 280°C, 230°C and 150°C, respectively. A Zebron capillary GC column of 30 mm x 0.25 mm, 0.25 μm film thickness was used (ZB-1, Phenomenex, Torrance, USA). The GC oven was programmed as follows: 40°C held for 0 min, increased to 70°C at 5°C/min, held at 70°C for 3.5 min, increase to 160°C at 20°C/min, increased to 280°C at 35°C/min and finally held at 280°C for 3 min with a total run time of 20.43 min. The flow was set a 1.0 ml/min with helium as carrier gas. Data processing was carried out with MassHunter Workstation Software (MassHunter, Agilent Technologies).

## Fecal human microbiota analysis

### Metagenomics of fecal samples

Taxonomy of the fecal microbiome was characterized in a high-resolution fashion using whole-genome metagenomic shotgun sequencing (MGS). Microbial DNA extraction from frozen fecal samples and MGS sequencing using the Illumina HiSeq platform was performed as described previously.^55^ Genomic library preparation was performed using the Nextera XT Library preparation kit. Trimmomatic (v.0.32) was used to remove adapters and trim the ends of metagenomic reads.^49,56^ Cleaned metagenomic reads were processed through a previously published bioinformatics pipeline.^49^ Taxonomic compositions were profiled using the software tool MetaPhlAn2 and were expressed as relative abundances in the microbiome samples.^57^

### Metabolomics of fecal samples

Human data was obtained from the LifeLines-DEEP study cohort. In short, this cohort comprises 1,500 participants from the northern parts of the Netherlands for which multiple data layers (genetics, microbiome, phenotypes, etc.) are available. In this cohort, 255 participants’ fecal metabolites were measured. Levels of specific metabolites of interest (fructose, glucose and butyrate) in fecal samples were extracted from an untargeted Ultrahigh Performance Liquid Chromatography-Tandem Mass Spectroscopy (UPLC-MS/MS) analysis performed by Metabolon®, Inc., Morrisville, NC, USA. Full methodological details on the complete procedure can be found elsewhere.^58^ In brief, fecal samples were lyophilized and extracted at a constant per-mass basis. Recovery standards were added prior to the first step in the extraction process for quality-control (QC) purposes. Proteins and macromolecule derivatives were removed from the samples using methanol precipitation under vigorous shaking for 2 min (Glen Mills GenoGrinder 2000). Next, samples were split into five different fractions: two fractions for analysis by two separate reverse phases (RP)/UPLC-MS/MS methods with positive ion mode electrospray ionization (ESI), one fraction for analysis by RP/UPLC-MS/MS with negative ion mode ESI, one fraction for analysis by HILIC/UPLC-MS/MS with negative ion mode ESI, and one fraction reserved as back-up. Metabolites profiles were derived from untargeted metabolomic experiments and each metabolite was expressed as a relative proportion represented as areas under the curve (AUC) of mass-spectrometry experiments, therefore, we could not estimate the total concentrations of fructose or glucose present in the fecal samples.

## Quantification and statistical analysis

### Metabolomics of fecal samples

Three types of controls were used and analyzed together with all experimental samples: 1) a pooled matrix sample, generated by extracting a small volume of each experimental sample, and serving as technical replicate throughout the dataset, 2) extracted water samples serving as process blanks, and 3) a cocktail of QC standards. Metabolites were identified by comparison to a reference library containing chemical standards. Area under the curve (AUC) analysis was performed for peak quantification and normalized to day median values. Control and curation procedures were performed to ensure high quality of the data and true chemical assignment, while removing artifacts and background noise. A total of 1,192 metabolites were measured in 255 fecal samples.

Metabolome values were log-10 transformed and centered around the mean (mean = zero and standard deviation = 1). Metabolomic values below the lower limit of detection (LLoD) were transformed as half of the lowest value observed for each individual metabolite. Regarding the metagenomic data, gut bacteria were analyzed at species-level. Bacterial species present in less than 20% of fecal samples were removed. Relative abundances of bacterial species were arcsine square root-transformed before entry into analysis.

### Statistical analyses

Data analysis and data visualization were performed using GraphPad Prism® (GraphPad, version 8.0), with experimental data expressed as means (μ) ± standard deviations (SD). Different conditions were compared using the two-way ANOVA test with Bonferroni post-hoc comparisons for the difference between two separate conditions. *P*-values ≤ 0.05 were considered statistically significant.

## Supplementary Material

Supplemental Material
