# Supplementary material for: Inulin-grown Faecalibacterium prausnitzii cross-feeds fructose to the human intestinal epithelium
Source: Gut Microbes. 2021 Nov 18;13(1):1993582. doi: 10.1080/19490976.2021.1993582 (PMC8604389; doi:10.1080/19490976.2021.1993582)
Supplement: Supplemental Material [file KGMI_A_1993582_SM2156.zip › Supplementary information/Supplementary Figures captions.docx]

**Supplementary Figure S1. HoxBan coculture system with glucose depleted from the bacterial compartment, but with glucose DMEM. (A-D)**. Effect of glucose depletion in the bacterial compartment of the HoxBan coculture system on *F. prausnitzii*-regulated expression of *NOS2* (inflammation marker, **A**), *HMOX1* (oxidative stress marker, **B**) and *IL1B* (inflammation marker, **C**) in Caco-2 cells. **(D)** Glucose-grown and glucose-depleted *F. prausnitzii* in the absence and presence of Caco-2 cells. In the absence of Caco-2 cells (top panels), *F. prausnitzii* growth is visible in the upper part of the bacterial compartment forming a rim below the oxic-anoxic interphase (black arrow). In the presence of Caco-2 cells (bottom panels), *F. prausnitzii* growth is enhanced (red arrow) and colonies appear more abundant and closer to the oxic-anoxic interphase where the Caco-2 cells reside (black arrow). All experiments were performed with two biological replicates, each with an *N* = 3.

**Supplementary Figure S2**. **Transient fructose accumulation from *in vitro* stool fermentation of inulin.** Diluted stool sample (1:1000) was inoculated anaerobically in YFCAI medium and (**A**) monosaccharides (fructose in purple and glucose in green) and (**B**) pH were measured at time points up to 48 h. Experiment performed in duplicate (*n* = 1). Glucose and fructose concentrations observed at t=0 are considered contaminations of the original inulin stock. While glucose only decreases after t=0, fructose levels transiently rise at t=2 and t=8.

**Supplementary Figure S3. Fecal glucose levels positively correlate with *F. prausnitzii* abundance.** **(A)** Volcano plot showing bacterial species whose relative abundance correlates significantly with fecal glucose levels in a population cohort (n=255). *E. rectale* levels most strongly correlate positively with fecal glucose levels, followed by *F. prausnitzii* levels (*R* = 0.28, *p_(fdr)_* = 0.0002; **B**), while *A. muciniphila* levels most strongly correlate negatively with fecal glucose levels.
