# Supplementary material for: Inulin-grown Faecalibacterium prausnitzii cross-feeds fructose to the human intestinal epithelium
Source: Gut Microbes. 2021 Nov 18;13(1):1993582. doi: 10.1080/19490976.2021.1993582 (PMC8604389; doi:10.1080/19490976.2021.1993582)

Supplementary Figure S1.

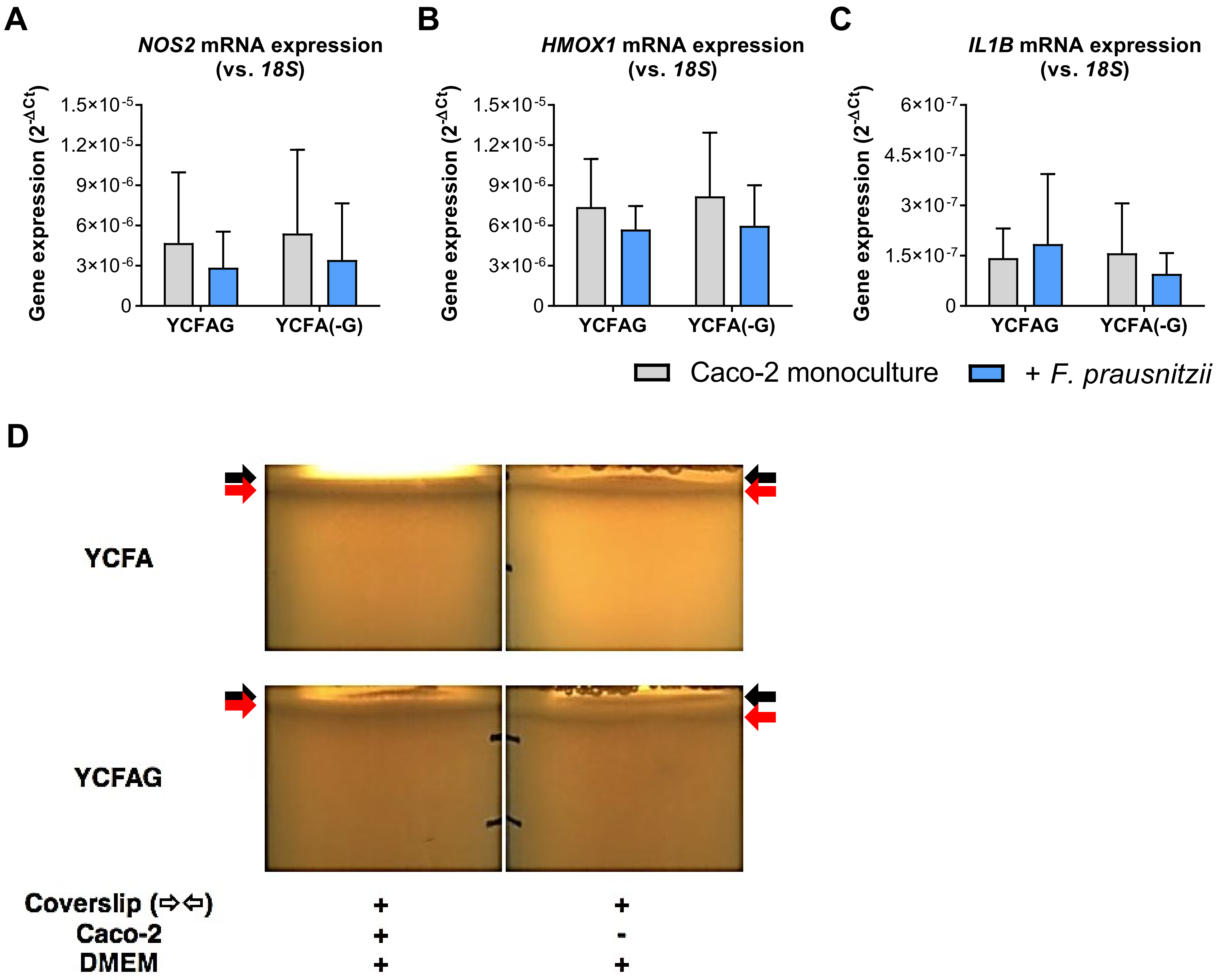

Supplementary Figure S2.

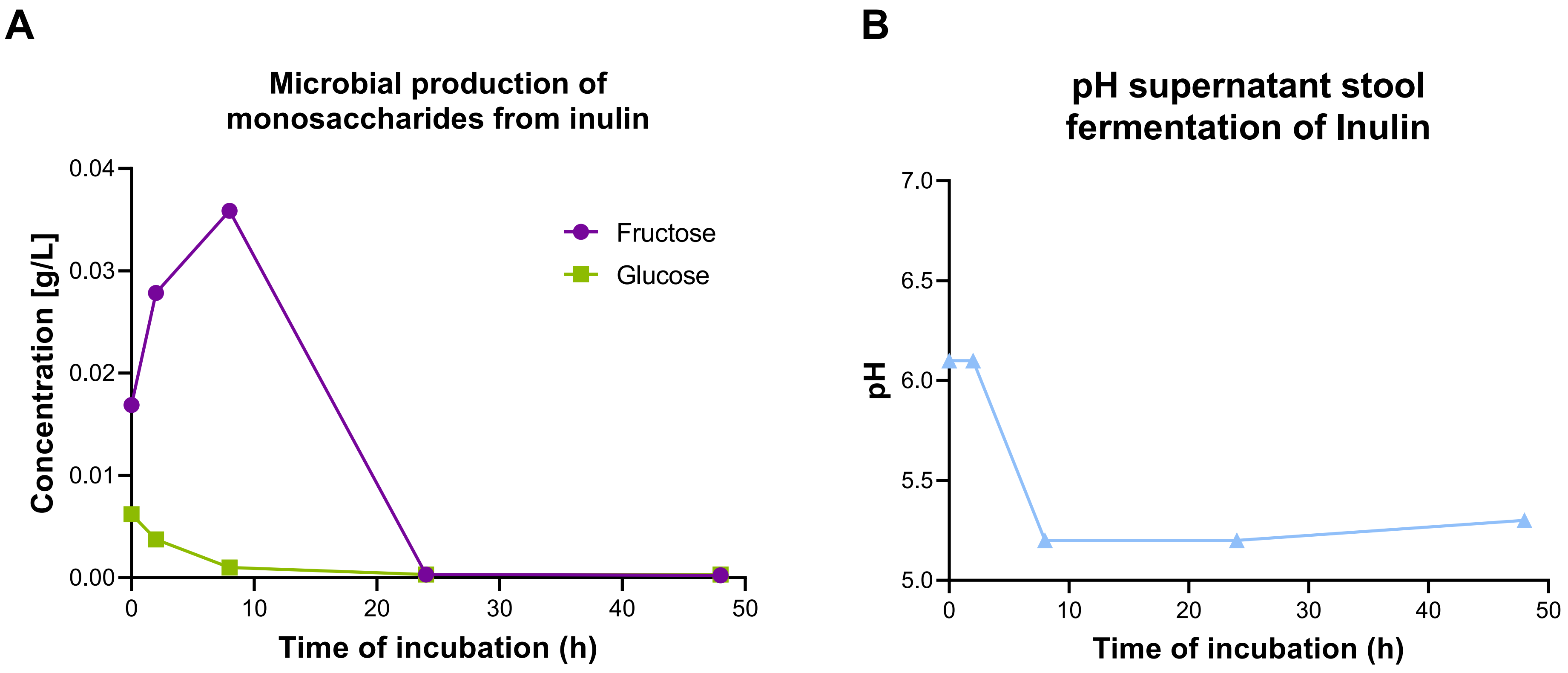

Supplementary Figure S3.

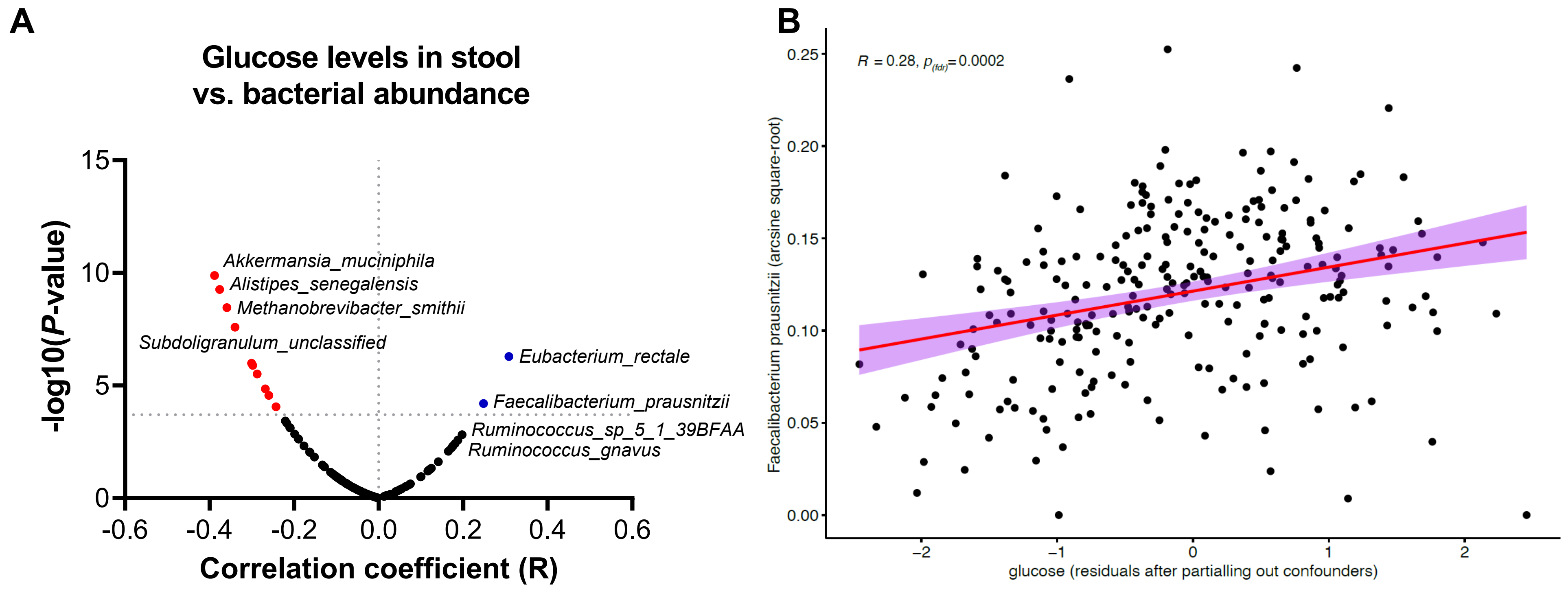

Supplement: Supplemental Material [file KGMI_A_1993582_SM2156.zip › Supplementary information/supplementary figures.pdf]
